# Evaluation of anticancer effects of *Juniperus communis* extract on hepatocellular carcinoma cells *in vitro* and *in vivo*

**DOI:** 10.1042/BSR20211143

**Published:** 2021-07-12

**Authors:** Nan-Chieh Huang, Ru-Lai Huang, Xiao-Fan Huang, Kai-Fu Chang, Chien-Ju Lee, Chih-Yen Hsiao, Shan-Chih Lee, Nu-Man Tsai

**Affiliations:** 1 Department of Information Engineering, I-Shou University, Kaohsiung 84001, Taiwan, R.O.C.; 2 Division of Family Medicine, Zuoying Branch of Kaohsiung Armed Forces General Hospital, Kaohsiung 81342, Taiwan, R.O.C.; 3 Division of Endocrinology and Metabolism, Department of Internal Medicine, Ditmanson Medical Foundation Chia-Yi Christian Hospital, Chia-Yi 60002, Taiwan, R.O.C.; 4 Department of Medical Laboratory and Biotechnology, Chung Shan Medical University, Taichung 40201, Taiwan, R.O.C.; 5 Division of Nephrology, Department of Internal Medicine, Ditmanson Medical Foundation Chia-Yi Christian Hospital, Chia-Yi 60002, Taiwan, R.O.C.; 6 Department of Hospital and Health Care Administration, Chia Nan University of Pharmacy and Science, Tainan 71710, Taiwan, R.O.C.; 7 Department of Medical Imaging and Radiological Sciences, Chung Shan Medical University, Taichung 40201, Taiwan, R.O.C.; 8 Department of Medical Imaging, Chung Shan Medical University Hospital, Taichung 40201, Taiwan, R.O.C.; 9 Clinical Laboratory, Chung Shan Medical University Hospital, Taichung 40201, Taiwan, R.O.C.

**Keywords:** Apoptosis, Cell cycle, Hepatocellular carcinoma, Juniperus communis, Natural product

## Abstract

Hepatocellular carcinoma (HCC) is the most common type of primary liver cancer and accounts for the fourth leading cause of all cancer deaths. Scientific evidence has found that plant extracts seem to be a reliable choice due to their multitarget effects against HCC. *Juniperus communis* has been used for centuries in traditional medicine and its anticancer properties have been reported. As a result, the purpose of the study was to investigate the anticancer effect and mechanism of *J. communis* extract (JCo extract) on HCC *in vitro* and *in vivo*. In the present study, we found that JCo extract inhibited the growth of human HCC cells by inducing cell cycle arrest at the G_0_/G_1_ phase, extensive apoptosis and suppressing metastatic protein expressions in HCC cells. Moreover, the combinational treatment of JCo and VP-16 was found to enhance the anticancer effect, revealing that JCo extract might have the potential to be utilized as an adjuvant to promote HCC treatment. Furthermore, *in vivo* study, JCo extract significantly suppressed HCC tumor growth and extended the lifespan with no or low systemic and pathological toxicity. JCo extract significantly up-regulated the expression of pro-apoptotic proteins and tumor suppressor p53, suppressed VEGF/VEGFR autocrine signaling, down-regulated cell cycle regulatory proteins and MMP2/MMP9 proteins. Overall, our results provide a basis for exploiting JCo extract as a potential anticancer agent against HCC.

## Introduction

Hepatocellular carcinoma (HCC) is the sixth most common cancer, making it a major health concern worldwide. Prevalent treatments for HCC include surgery, chemotherapy, radiation therapy, and targeted therapy [1]. Chemotherapy is usually used to promote the patients’ survival. However, chemodrugs are often associated with toxicity and strong side effects that impair therapeutic efficacy, resulting in high death rates [2]. Consequently, the discovery of novel antitumor agents with better therapeutic efficacy and less toxicity has become an attractive avenue for preventing and treating HCC. Natural products from plants may be a safer choice because use alone or a combination of medicinal plants and conventional therapies has a beneficial effect on survival, immune regulation, and quality of life [3].

The recent trends in drug development focus on natural resources, mainly medicinal plant parts, including leaves, roots, bark, fruits, and seeds are used in herbal medicine. Dietary intake of specific chemical compounds can reduce inflammation, oxidization, and the risk of cancer [4]. In particular, some aliments can affect cancer cells via modified regulation of the cell cycle, cell death, angiogenesis, and metastasis [5,6]. The induction of cell cycle arrest and apoptosis is the most promising approach for curbing tumor cell survival and progression. Indeed, several bioactive components have been reported to target these processes in order to produce anticancer or chemopreventive effects [7].

*Juniperus communis* has been extensively used in traditional medicine. Extract deriving from different parts of the *J. communis* tree have been proven to have anti-inflammatory [8,9], antidiabetic [10–12], antioxidant [13,14], antimicrobial activities [15,16] as well as anticancer properties [17–20]. Currently, the antitumor properties of *J. communis* extract (JCo extract) and the underlying molecular mechanisms remain unknown in the case of HCC. In the present study, we utilized JCo extract to determine their effects and mechanisms of action in the growth inhibition of human HCC cells.

## Materials and methods

### Preparation of *J. communis* plant extract

Extract of *J. communis* fruits which was obtained from Nepal was generated via steam distillation by PHOENIX (New Jersey, U.S.A.). After extraction, two layers were generated, one was an aqueous layer and another was an oil layer also called essential oil which we used throughout experiments. The JCo extract was preserved at 4°C in a brown glass bottle and sealed with parafilm to save the extract from moisture and light. The JCo extract was freshly dissolved in dimethyl sulfoxide (DMSO) to be used for the experiments. The drug concentration was calculated using the following formula: JCo extract (μg)/volume (ml).

### Cell culture conditions

HCC cells (HepG_2_, Mahlavu, and J5) and normal cells (MDCK and SVEC) were obtained from the Bioresource Collection and Research Center (BCRC) in Taiwan (Hsinchu, Taiwan). HepG_2_, Mahlavu, MDCK, and SVEC cells were cultured in Dulbecco’s modified Eagle’s medium (DMEM), while J5 cells were cultured in Roswell Park Memorial Institute (RPMI) 1640 medium supplemented with 10% fetal bovine serum, sodium pyruvate, HEPES and Penicillin/Streptomycin in a humidified atmosphere with 5% CO_2_ at 37°C. All cell culture reagents were purchased from Gibco/Thermo Fisher Scientific (Waltham, MA, U.S.A.).

### Cell viability assay

Cells cultured in a 96-well plate at a density of 5 × 10^3^ cells/well overnight; when they reached 50–60% confluence, they were treated with JCo extract and Etoposide (VP-16) for 24, 48 and 72 h. Cell viability was then determined using an MTT assay (Amresco, Radnor, PA). The MTT formazan dye was dissolved in DMSO and detected using Spectra Max plus 384 Microplate Reader (Molecular Devices, U.S.A.) at a wavelength of 550 nm. HepG_2_ and Mahlavu cells were analyzed with a Femtopath TP53 Exon8 Primer Set (HongJing Biotech., New Taipei City, Taiwan) to confirm their TP53 status.

### Evaluation of the combinatorial effect

The effects of combined treatments with JCo extract and VP-16 were evaluated through a combination index (CI). Growth inhibition curves were traced based on data from the MTT assay. The CI was calculated using CompuSyn software (ComboSyn, Inc., Paramus, NJ, U.S.A.) [21,22]. The significant differences between single drugs and drug combinations were tested via Student’s *t* test (*P*<0.05).

### Cell cycle analysis

Cells (2 × 10^6^) were treated with JCo extract (50 μg/ml) for the indicated time. After the treatment, cells were harvested and stained with Propidium Iodide (PI; 40 μg/ml) and RNase (1 mg/ml; Sigma, Missouri, MO, U.S.A.), and incubated overnight at 4°C with shaking. Cells were then resuspended in phosphate-buffered saline and collected. Ten thousand cells were analyzed by flow cytometry, and the percentage of cells assigned to each phase of the cell cycle was calculated using the CellQuest Pro software (BD, Franklin Lakes, NJ). The percentage of cells in the sub-G_1_ phase was calculated with the following formula: (number of cells in sub-G_1_ phase/total cell number) × 100%.

### TUNEL assay

Terminal deoxynucleotidyl transferase-mediated dUTP nick-end labeling (TUNEL) assay was performed using the TUNEL *in situ* cell death detection kit (Sigma, Missouri, MO) according to the manufacturer’s instructions. Cells were fixed with 10% formalin and exposed to 0.01% Triton X-100 for 2 min on ice. After that, cells were incubated with TUNEL reaction buffer. The nuclei of the cells were counterstained with PI (10 μg/ml) and examined under a fluorescence microscope. For tissue TUNEL staining, the slides were rehydrated, incubated with H_2_O_2_, and blocked with 10% bovine serum albumin (BSA, Sigma, Missouri, MO, U.S.A.). After blocking, the slides were incubated with TUNEL reaction buffer and PI (1 μg/ml) for 30 min. The number of TUNEL-positive cells was quantified upon observation of ten random fields under 200× magnification and analyzed via Photoshop (Adobe.Photoshop.CS3.Extended v10.0).

### Immunoblotting analysis

HepG_2_ cells (5 × 10^6^ cells/dish) were seeded in 10-cm cell culture dishes and treated with JCo extract (50 μg/ml) for the indicated time. Cells were harvested, and cell lysates were subjected to Western blot analysis according to a previously published protocol [23]. The intensity of protein expression was measured using the ImageJ software (NIH, Betlesda, MD, U.S.A.). Antibodies against p-p53, p-Rb, p21, CDK2, CDK4, cyclin A, cyclin B1, cyclin D1, Bax, Bcl2, FAS, FASL, caspase-3, caspase-8, caspase-9, VEGFA, VEGFR1, VEGFR2 and β-actin (1:200) were purchased from Santa Cruz Biotechnology (Dallas, TX).

### Animal study

HepG_2_ cancer cells (1 × 10^6^) were injected subcutaneously into the right flank of female BALB/c nude mice, which were purchased from the National Laboratory Animal Breeding and Research Center (Taipei, Taiwan). The experiment was performed at Laboratory Animal Center of Chung Shan Medical University (CSMU) and the procedure was approved by the Institutional Animal Care and Use Committee (IACUC) of CSMU (approval number: CSMU-IACUC-1662). After 5 days from the injection, the mice were randomly divided into two groups (five mice/group). Both groups were treated every 2 days with a vehicle (mineral oil, 100 μg/ml) by subcutaneous (s.c.) administration, while only the experimental group was also treated with JCo extract (200 mg/kg). After tumor size reached approx. 1500 mm^3^ (length × width × height), the mice were killed by carbon dioxide asphyxiation, and the collected tumors and organs were fixed in 10% formalin, embedded, and stained with Hematoxylin and immunohistochemical staining was performed. The quantitative score was calculated upon observation of ten random fields under 200× magnification and the pictures were analyzed using Photoshop (Adobe.Photoshop.CS3.Extended v10.0). The methods of staining and scoring were operated according to a previously published protocol [23].

### Statistical analysis

All results are expressed as the mean ± standard deviation (SD). Statistically significant differences between groups were determined via Student’s *t* test or one-way ANOVA. A *P*-value <0.05 was considered as the threshold for statistical significance.

## Results

### JCo extract significantly inhibited the growth of HCC cells

**Figure 1:**
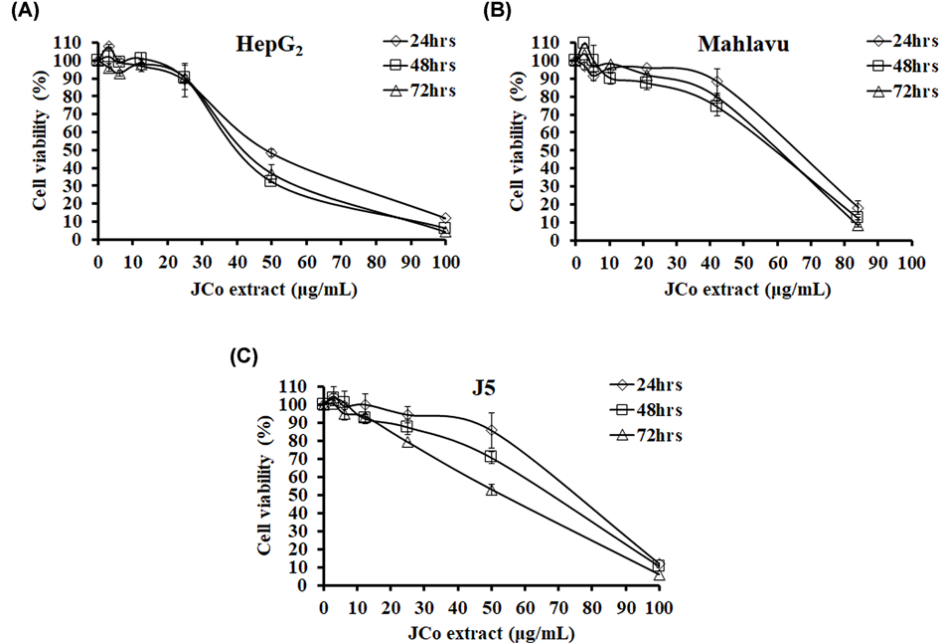
Growth inhibitory effects of JCo extract in HCC cells Three different lines of HCC cells (HepG_2_, Mahlavu, and J5) were seeded in 96-well plates. HepG_2_ cells (**A**), Mahlavu cells (**B**), and J5 cells (**C**) were treated with serial concentrations (0–200 μg/ml) of JCo extract for 24, 48 or 72 h. After treatment, cell viability was measured by MTT assay, as described in the ‘Materials and methods’ section. Data are expressed as the mean ± SD biological replicates.

To assess the anticancer properties of JCo extract, their inhibitory effects on the growth of HCC cells were first determined by MTT assay. HCC cells were treated with various concentrations of JCo extract (0–100 μg/ml) for different time spans. As shown in Figure 1, treatments with JCo extract caused dose-dependent growth inhibition of HCC cells. To determine the toxicity of JCo extract on normal cells, their growth inhibitory effects on kidney epithelial and vascular endothelial cells were determined by MTT assay. As shown in Table 1, treatments with JCo extract did not lead to significant growth inhibition in either of the two normal cell lines. Furthermore, the inhibitory effects of VP-16, a clinical drug, were also tested. The results showed that VP-16 had greater inhibitory effects on normal cells than JCo extract (Table 1). These results indicated that the JCo extract strongly inhibited the growth of HCC cells and had higher selection against tumor cells than normal cells.

**Table 1:** The IC_50_ of JCo extract and VP-16 in HCC and normal cells

		JCo extract (μg/ml)			VP-16 (μg/ml)		
Cell line	Tumor type	24 h	48 h	72 h	24 h	48 h	72 h
HCC cells							
Mahlavu	Human HCC	64.9 ± 3.1*^1,2^*	58.5 ± 2.3*^1,2^*	59.4 ± 1.0*^1,2^*	54.7 ± 7.0	5.5 ± 0.8	<6.25
HepG2	Human HCC	48.9 ± 1.5*^1,2^*	42.3 ± 0.2*^1,2^*	43.9 ± 2.6*^1,2^*	>200	18.1 ± 2.8	<6.25
J5	Human HCC	74.2 ± 3.6*^1,2^*	67.2 ± 2.0*^1,2^*	53.2 ± 3*^1,2^*	>200	14.1 ± 0.9	7.3 ± 2.7
Normal cells							
MDCK	Canis kidney epithelial cell	>87.7	>87.7	84.0 ± 0.6	>50	25.7 ± 2.7	2.7 ± 0.2
SEVC	Mouse vascular endothelia cell	78.8 ± 0.4	67.4 ± 0.4	66.8 ± 0.05	21.7 ± 4.9	1.9 ± 0.06	1.8 ± 0.1

Values are reported as mean ± SD (μg/ml). It was significantly different that compared HCC cells with MDCK (1) or SVEC (2) in JCo extract treatment at the same time points (*P*<0.05).

### JCo extract enhanced the growth inhibition of VP-16 in HepG_2_ cells

**Figure 2:**
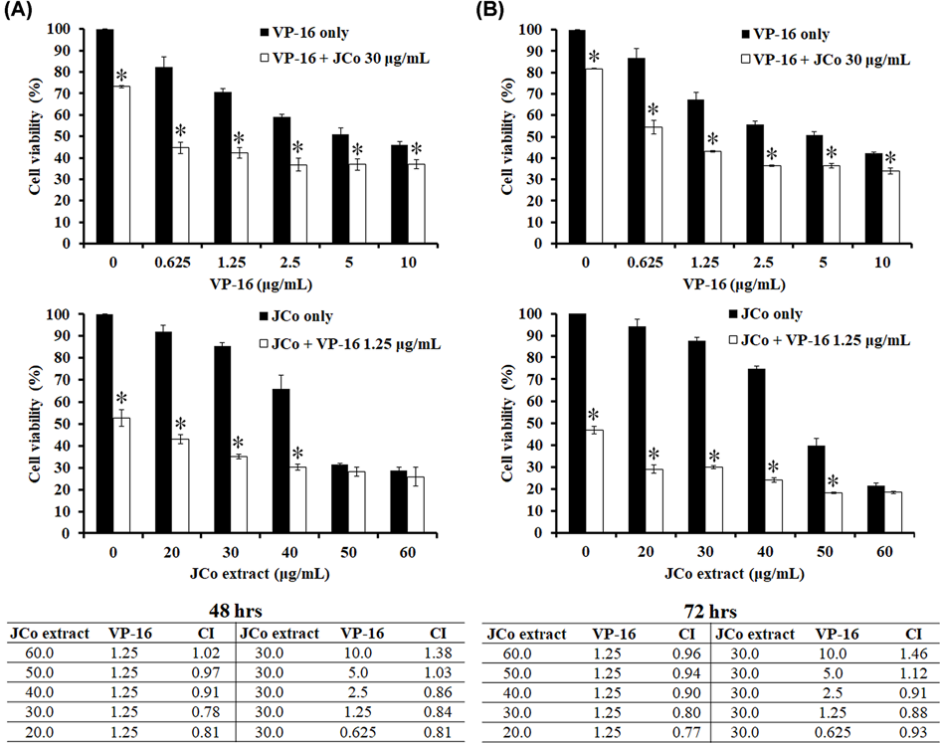
Effects of JCo extract/VP-16 combinations on HepG_2_ cell growth HepG_2_ cells were seeded in 96-well plates and treated with the indicated drug concentrations for the indicated time. The viability and CI values of HepG_2_ cells were measured after treatment for 48 h (**A**) or 72 h (**B**). Results are expressed as percentages of cell viability (mean ± SD). *: significant differences between combined and single treatments (*P*<0.05).

To determine the effect of combined treatment with JCo extract and VP-16, the cell viability was determined by MTT assay for both drugs alone and their combination, and a CI was calculated. HepG_2_ cells were seeded in 96-well plates treated with one drug alone (JCo extract or VP-16) and a drug combination for 48 and 72 h, and the rate of growth inhibition was compared between each drug alone and the drug combination. As shown in Figure 2, 0.625–10 μg/ml of VP-16 plus 30 μg/ml of JCo extract significantly reduced the cell viability, ranged from 54.39 ± 3.29 to 33.86 ± 1.52% and 20–60 μg/ml of JCo extract plus 1.25 μg/ml of VP-16 statistically decreased cell viability, ranged from 42.98 ± 2.16 to 18.24 ± 0.33%. The results revealed that combined VP-16/JCo extract treatment showed stronger inhibitory effects at 48 and 72 h than VP-16 or JCo extract alone. After that, the CI was less than 1 while JCo extract (≤40 μg/ml) combined with VP-16 (≤2.5 μg/ml), suggesting that the JCo extract and VP-16 acted synergistically in cell growth inhibition and the combination formulas reduced the concentration of VP-16 and JCo extract. It indicated that the combination of JCo extract and VP-16 not only enhanced the inhibitory effect of VP-16 but also might be reduced side effects potentially.

### JCo extract induced cell cycle arrest and apoptosis, and reduced expression of proteins involved in tumor autocrine signaling

**Figure 3:**
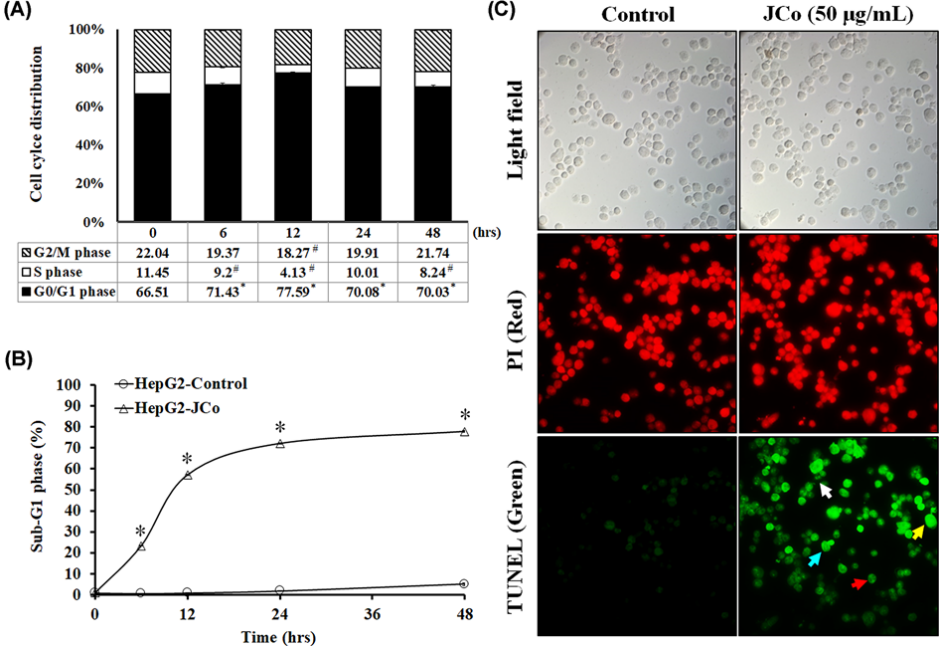
Effects of JCo extract on cell cycle distribution and apoptosis in HepG_2_ cells HepG_2_ cells were seeded in 96-well plates and then treated with JCo extract (50 μg/ml) for 0, 6, 12, 24 or 48 h. (**A**) Analysis of cell cycle distribution of cells treated with JCo extract by flow cytometry. (**B**) Cells in the sub-G_1_ phase are presented as a percentage of the total cell count. (**C**) After treatment with JCo extract, cells were harvested and stained by TUNEL. Red: PI; green: TUNEL positive; white arrow: apoptotic bodies; yellow arrow: DNA fragments; blue arrow: chromatin condensation; red arrow: anoikis. *: significant increases in treated cells as compared with control cells; #: significant decreases in treated cells as compared with control cells (*P*<0.05).

The cell cycle can be divided into G–G_1_, S and G_2_–M stages, which are considered to be of great significance for controlling tumor growth. Next, to investigate the molecular mechanisms by which JCo extract inhibited the growth of HepG_2_ cells, cell cycle analysis was performed. HepG_2_ cells were treated with JCo extract at 50 μg/ml for the indicated time. As shown in Figure 3A, drug treatment increased the percentage of cells in G_0_/G_1_ phase while reducing the percentage of cells in the S-phase as compared with mock treatment. In addition, the cell population in the sub-G_1_ phase significantly increased upon drug treatment in a time-dependent manner (Figure 3B). Apoptosis plays a vital role in eliminating mutant or excessively proliferating cells in the system to balance the natural environment and interfere with tumor growth. Therefore, it is considered to be a protective mechanism to prevent tumor progression. Next, to assess changes in the apoptotic morphology of cells treated with JCo extract, a TUNEL assay was performed. The results showed that JCo extract induced apoptosis in HepG_2_ cells (Figure 3C). The results suggested that JCo extract induced cell cycle arrest at G_0_/G_1_ phase and cell apoptosis in HCC cells.

**Figure 4:**
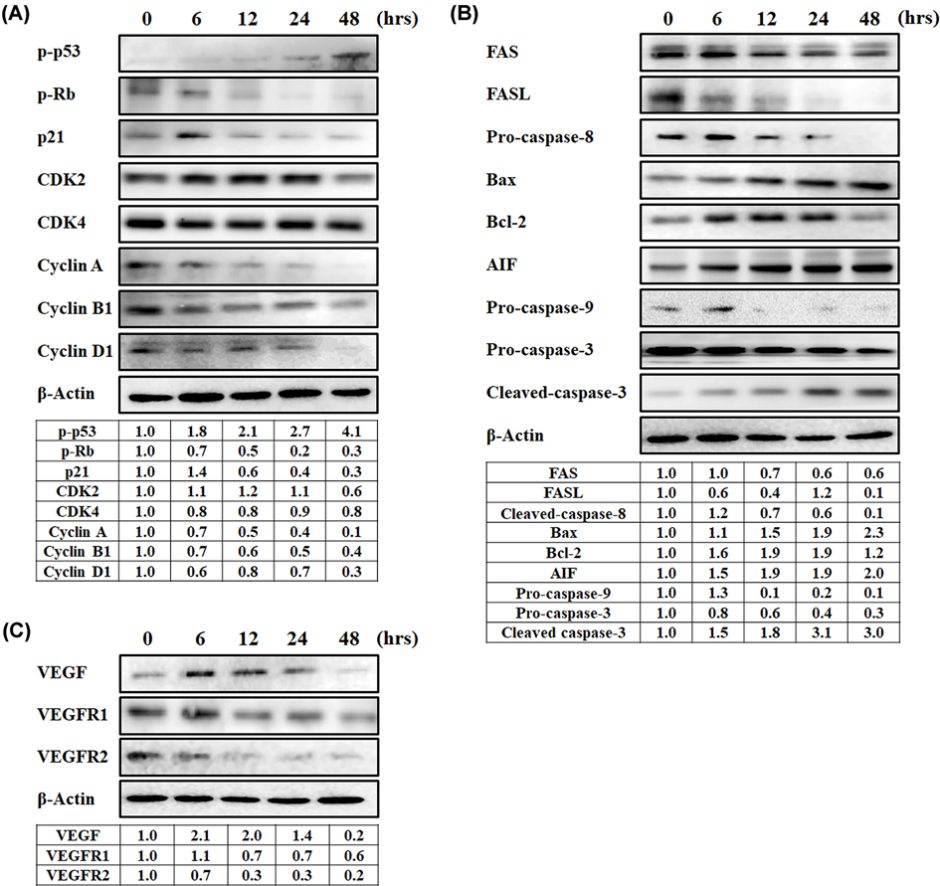
Effects of JCo extract on the levels of proteins involved in cell cycle, apoptosis and autocrine signaling in HepG_2_ cells Cells (2 × 10^6^ cells) were seeded in 10-cm cell culture dishes and treated with JCo extract (50 μg/ml) for the indicated time and then harvested. Total protein content was quantified by Western blot and measured using the ImageJ software. (**A**) Cell cycle-related proteins; (**B**) apoptosis-related proteins; (**C**) VEGFA/VEGFR proteins.

To further understand the specific mechanism of JCo extract in causing cell cycle arresting in HepG_2_ cells, the expression of cell cycle-associated proteins was evaluated using Western blotting. After treatment with JCo extract, the levels of p-p53 increased along with p21, while the level of p-Rb was reduced; besides, the levels of cell cycle regulators, including CDK2, CDK4, cyclin A, cyclin B1, and cyclin D1 decreased in a time-dependent fashion (Figure 4A). In order to further clarify the molecular mechanisms underlying the induction of apoptosis in HepG_2_ cells by JCo extract, the total protein extract was collected and analyzed by Western blot. The levels of FAS, FASL, and procaspase 8 were reduced after treatment with JCo extract. Moreover, the levels of BAX and AIF increased while those of BCL2 and procaspase 9 decreased, and were accompanied by a reduction in procaspase 3 levels and increment of cleaved caspase-3 levels (Figure 4B). It indicated that JCo extract activated extrinsic and intrinsic caspase cascades, leading to cleavage of caspase-3 and the induction of cell apoptosis. Furthermore, the effects of JCo extract on the modulation of autocrine signaling were also examined by Western blot. The results showed that JCo extract inhibited the expression of VEGFA, VEGFR1 and VEGFR2, suggesting that JCo extract inhibited autocrine signaling (Figure 4C). Taken together, our results demonstrated that JCo extract induced cell cycle arrest, apoptosis, and inhibited tumor autocrine signaling, resulting in growth inhibition of HCC cells.

### JCo extract suppressed tumor growth of HCC *in vivo*

**Figure 5:**
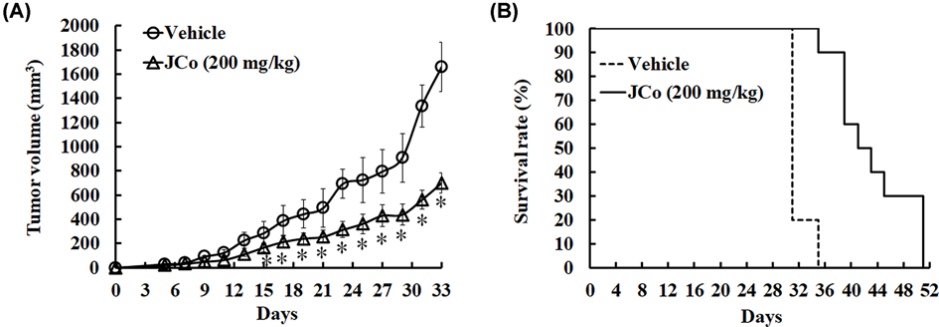
Effects of JCo extract on tumor growth in tumor-bearing nude mice HepG_2_ cells were subcutaneously injected into the right flank of female nude mice (*n*=3). Mice were divided into an experimental and a control group (*n*=5), and both groups were subcutaneously injected with mineral oil (100 μg/ml), while only the experimental group also received JCo extract (200 mg/kg). Tumor volumes were determined every 2 days. (**A**) Tumor volume (mm^3^); (**B**) survival rate (%). *: significant differences between the experimental and control group (*P*<0.05).

**Figure 6:**
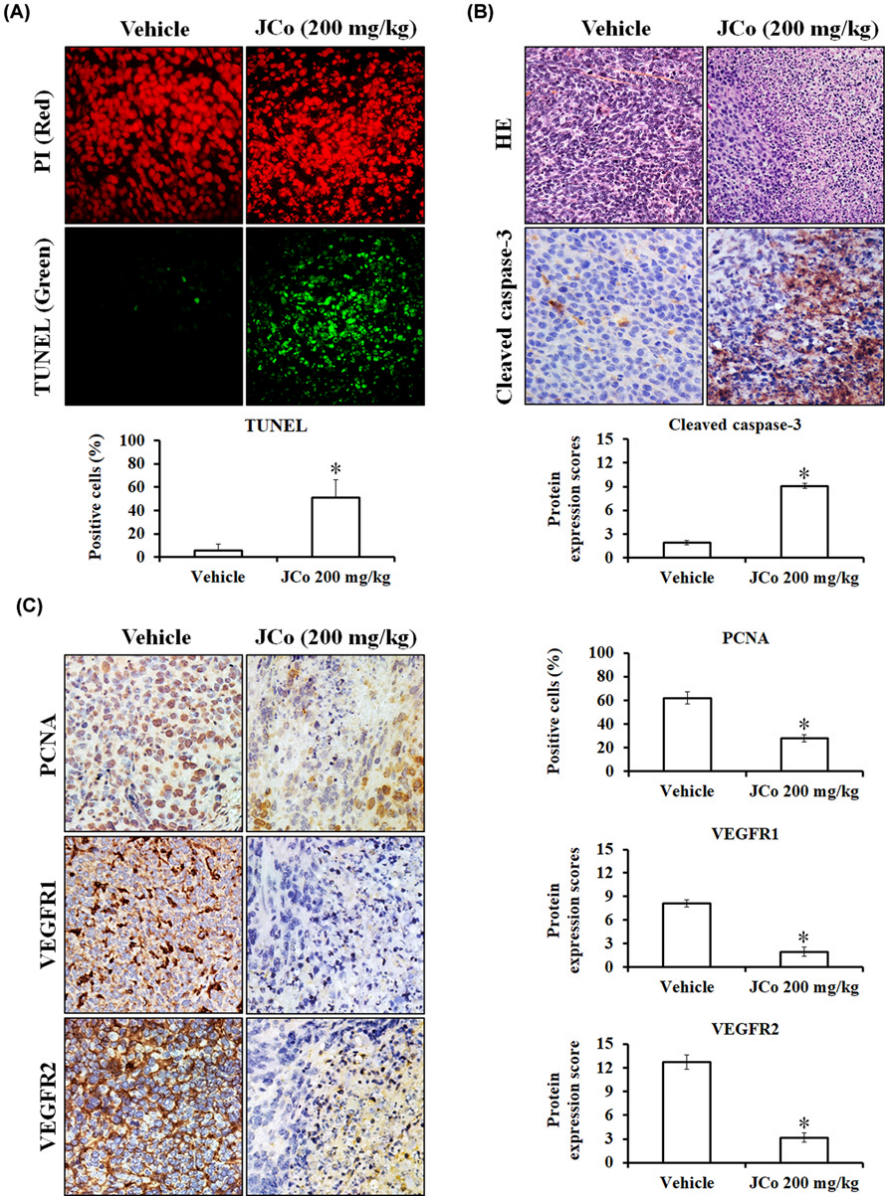
The anticancer mechanisms of JCo extract on HCC *in vivo* (**A**) Tumor tissue sections were rehydrated and stained with TUNEL. Red: PI; green: TUNEL positive. (**B**) H&E and immunohistochemical analysis of tumor morphology (×200) and cleaved caspase 3 (×400) in tumors treated or not with JCo extract. (**C**) Immunohistochemical analysis of PCNA, VEGFR1 and VEGFR2 content in tumors treated or not with JCo extract. The quantitative score was calculated upon observation of ten random fields under 200× magnification. *: significant differences between treated and untreated tissues (*P*<0.05).

The above experiments demonstrated that JCo extract inhibited the proliferation of HCC cells and promoted apoptosis *in vitro*. Based on these findings, we studied the effect of JCo extract on tumor growth in BALB/c (nu/nu) nude mice injected s.c. with HepG_2_ cells. The experimental group was treated with JCo extract every 2 days, while the control group was treated only with a mock vehicle until tumor size reached approximately 1500 mm^3^. Mice were then killed, and collected tumors and organs were subjected to histological analysis. Treatment with JCo extract suppressed HCC tumor growth (Figure 5A) and significantly prolonged the lifespan of treated mice (51 days) as compared with control mice (37 days) (Figure 5B). To verify the antitumor properties of JCo extract *in vivo*, the effects of JCo extract on apoptosis and proliferation of mice tumor cells were elucidated. Tumor mass staining via TUNEL revealed a great number of apoptotic cells in organs of mice treated with JCo extract (51 ± 15.23%), indicating that JCo extract led to apoptosis of tumor cells (Figure 6A). Tumor morphology was observed after H&E staining and showed that JCo extract caused the death of tumor tissue accompanied by increased accumulation of cleaved caspase 3 protein (Figure 6B). Moreover, the levels of PCNA, VEGFR1 and VEGFR2 were reduced, indicating that JCo extract inhibited tumor proliferation and autocrine signaling (Figure 6C). These results demonstrated that JCo extract suppressed tumor growth *in vivo* via inhibition of proliferation and induction of apoptosis, consistent with *in vitro* results.

### JCo extract reduced the expression of angiogenesis and metastasis-related proteins *in vivo*

**Figure 7:**
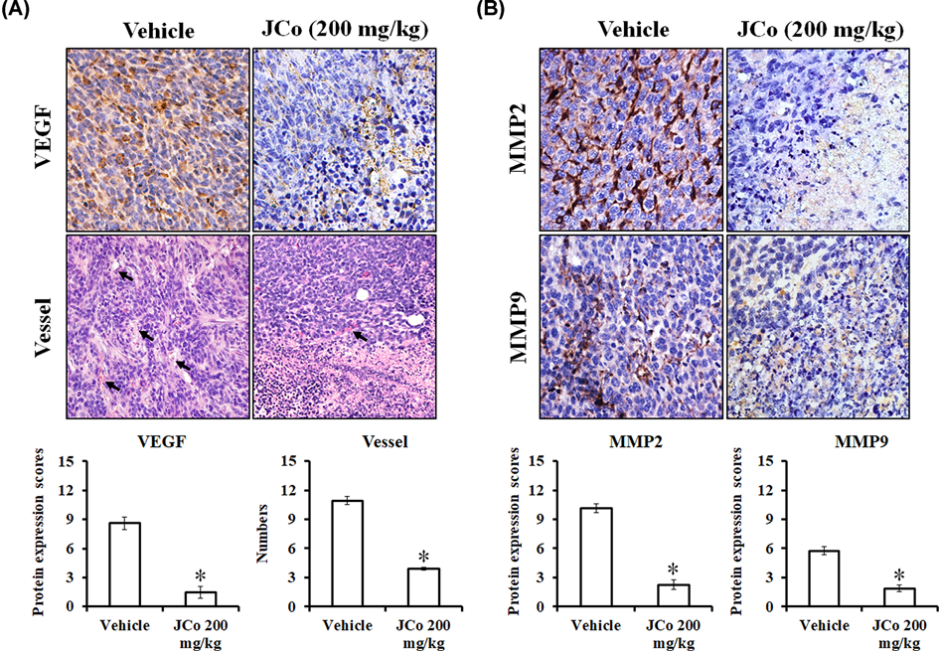
Inhibitory effects of JCo extract on angiogenesis and metastasis of HCC *in vivo* (**A**) Immunohistochemical and H&E analysis of VEGFA content and tumor vessel number in tumors treated or not with JCo extract. Arrows indicated blood vessels. (**B**) Immunohistochemical analysis of MMP2 and MMP9 content in tumors treated or not with JCo extract. The quantitative score was calculated upon observation of ten random fields under 200× magnification. *: significant differences between treated and untreated tissues (*P*<0.05).

To examine the effect of JCo extract on angiogenesis and metastasis, the levels of VEGFA, MMP2 and MMP9 in the tumor tissue of treated mice were evaluated by IHC staining. The results showed that JCo extract not only inhibited VEGFA expression but also reduced vessel numbers (Figure 7A). On the other hand, JCo extract possessed antimetastatic potential since it reduced MMP2 and MMP9 expression (Figure 7B).

### The toxicity of JCo extract *in vivo*

**Figure 8:**
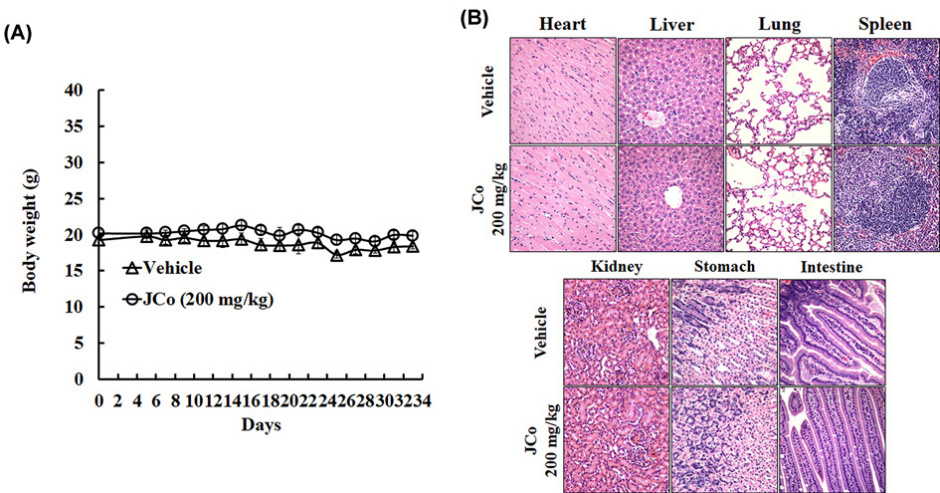
Analysis of pathological toxicity after treatment with JCo extract (**A**) HepG_2_ cells were subcutaneously injected into the right flank of female nude mice (*n*=3). Mice were divided into an experimental and a control group (*n*=5), and both groups were subcutaneously treated with mineral oil (100 μg/ml), while only the experimental group also received JCo extract (200 mg/kg). Body weights were determined every 2 days. (**B**) After the killing of mice, organs were collected, fixed with 10% formalin, and stained with H&E to observe the tissue morphology under 400× magnification.

To evaluate the tolerance to the toxicity of JCo extract in an HCC established animal model, we measured body weight and evaluated pathological toxicity in control mice as well as mice treated with JCo extract. JCo extract did not significantly affect the body weight of mice (Figure 8A). Besides, JCo extract also exhibited low or no pathological toxicity *in vivo* (Figure 8B). These results suggested that JCo extract showed low or no short-term acute toxicity or long-term accumulative toxicity *in vivo*, suggesting the therapeutic course of JCo extract was well-tolerated.

## Discussion

HCC is the sixth most common type of cancer in the world and the third most common cause of cancer-related deaths worldwide [1]. Systemic chemotherapy, such as sorafenib and lenvatinib, is used to treat patients with advanced HCC related to vascular invasion and metastasis. However, the median survival time of sorafenib treatment is 12.3 months, and the median survival time of levatinib treatment is 13.6 months [24], suggesting the long-term survival rate is still not satisfactory. Therefore, novel treatment strategies for HCC are required to increase the survival rates of patients. Natural products from plants may be a safer choice and have a beneficial effect on survival, immune regulation and quality of life [3]. In this study, we demonstrated the anticancer properties of JCo extract *in vitro*. JCo extract inhibited the growth of HCC cells in a dose-dependent manner. Also, as compared with the clinical drug VP-16, JCo extract exhibited lower cytotoxicity in normal cells, suggesting that JCo extract might induce fewer side effects after treatment. Moreover, JCo extract not only exerted inhibitory effects on HCC cells but also showed an enhancement of inhibition in combination with VP-16 *in vitro*, suggesting the potential application of JCo extract as adjuvant agents. Also, our results showed that the anticancer capacity of JCo extract was determined by the induction of cell cycle arrest via the activation of the p53/p21 pathway [25,26], repressed expression of cell cycle regulators and the activation of intrinsic and extrinsic apoptosis pathways via the caspase cascade [27–29]. On the other hand, we also found that JCo extract inhibited the protein expressions of VEGF, VEGFR1 and VEGFR2, which might block the autocrine or paracrine signaling of HCC [30]. Thus, the extracts of *J. communis* plant exerted antiproliferative effects through the induction of cell cycle arresting and apoptosis in HepG_2_ cells might serve as a potential anticancer agent for HCC.

Because it is difficult to cure HCC, prevention medicine might be an effective way to reduce cancer mortality. In our preliminary animal study, JCo extract was given a subcutaneous injection to inhibit HCC tumor growth and the results had shown that subcutaneous injection revealed the best therapeutic effect than oral gavage and intravenous injection. Moreover, JCo extract contains small molecules and lipophilic property compounds that might be slowly released into blood through subcutaneous tissue absorption and maintained drug concentration in the blood to continuously suppress tumor growth. Besides, our previous study showed that JCo extract exhibited well-suppressive effects on the growth of melanoma by subcutaneous treatment in C57/BL6 mice [23]. So, in the present study, we have chosen the subcutaneous injection to administrate JCo extract *in vivo*. The data showed that JCo extract suppressed tumor growth *in vivo*, improved the survival rate, and prolonged the lifespan of tumor-bearing mice, suggesting that JCo extract might be exploited as chemopreventive agents for preventing HCC progression. To get insights into the anticancer mechanisms *in vivo*, we examined tumor morphology after treatment with JCo extract, and we found that JCo extract induced apoptosis in tumor cells. Moreover, it has been found that HCC cells can express VEGF and VEGFR to trigger cell proliferation, survival, migration and invasion; besides, VEGF/VEGFR signaling can affect the prognosis and average survival time of patients [31,32]. Therefore, VEGF/VEGFR signaling can be one of a target for HCC treatment. In the results, JCo extract suppressed VEGF/VEGFR protein expressions *in vivo* that was consistent with the data found *in vitro*, suggesting JCo extract might reduce the autocrine and paracrine signaling pathways. Moreover, JCo extract reduced the vessel formation and suppressed MMP2/MMP9 protein expression *in vivo*, revealing that JCo extract might exert antimetastatic potential on HCC. Next, we also evaluated the toxicity of JCo extract *in vivo* by body weight and pathological analysis, and the results showed no significant difference in body weight after treatment with JCo extract. After pathological analysis, organ tissue showed no obvious damage after administration of JCo extract. As a result, JCo extract might display low or no toxicity to organs, suggesting JCo extract exhibited antihepatoma properties with well-tolerance.

To identify the major components with anticancer properties, JCo extract were analyzed using GC-MS in our previous study [23], they were including α-pinene (34.87%), citronellyl acetate (14.26%), limonene (10.72%), terpinolene (10.65%), *p*-cymene (6.21%), elemene (3.32%), cadinene (2.12%), cyclohexane (1.79%), cedrol (1.42%), caryophyllene (1.39%) and others (13.25%). Studies demonstrated that α-pinene inhibits cell growth in prostate cancer and ovarian cancer [33,34], and induces G_2_/M cell cycle arrest through up-regulation of Chk1 and Chk2 levels and down-regulation of cyclin B1, CDC25 and CDK1 levels in hepatoma carcinoma BEL-7402 cells [35]. d-Limonene stimulates autophagic flux in HepG2 and MCF7 cells [36] and shows chemoprevention of hepatocarcinogenesis in AKR mice via inhibition of c-jun and c-myc [37]. Terpinolene down-regulates AKT1 expression in K562 cells [38] and reveals inhibitory effects in N2a neuroblastoma cells [39]. *p*-Cymene significantly suppresses colorectal cancer occurrence in hyperlipidemic rats by decreasing the expression of serum inflammatory cytokines and intestinal oxidative-stress cytokines [40]. Other components (<5%) in JCo extract also have been reported the anticancer activities, such as elemene, cadinene, cedrol and caryophyllene. Therefore, these studies further confirm the anticancer potential of JCo extract due to a variety of anticancer components, thereby inhibiting cell proliferation and inducing cell apoptosis in HCC.

In conclusion, JCo extract exhibited anticancer activity with growth inhibition, repression of autocrine and paracrine signaling, and induction of cell apoptosis *in vitro* and *in vivo*. In light of the above safe and effective antitumor effects, JCo extract may represent a potential agent for the treatment of HCC patients.

## Data Availability

All data generated or analyzed during the present study are included in this article.

## Abbreviations

CI: combination index
DMSO: dimethyl sulfoxide
H&E: hematoxylin and eosin
HCC: hepatocellular carcinoma
IHC: immunohistochemistry
JCo extract: *J. communis* extract
MMP: matrix metalloproteinases
PI: propidium iodide
TUNEL: terminal deoxynucleotidyl transferase-mediated dUTP nick-end labeling
VEGF: vascular endothelial growth factor
VEGFR: vascular endothelial growth factor receptor
